# 6-Meth­oxy-9-phenyl­sulfonyl-2-(2-thien­yl)-9*H*-thieno[2,3-*b*]carbazole

**DOI:** 10.1107/S1600536809003493

**Published:** 2009-02-06

**Authors:** G. Chakkaravarthi, A. Marx, V. Dhayalan, A. K. Mohanakrishnan, V. Manivannan

**Affiliations:** a Department of Physics, CPCL Polytechnic College, Chennai 600 068, India; b Department of Physics, Presidency College, Chennai 600 005, India; c Department of Organic Chemistry, University of Madras, Guindy Campus, Chennai 600 025, India

## Abstract

In the title compound, C_25_H_17_NO_3_S_3_, the mean planes of the thieno[2,3-*b*]carbazole and phenyl rings are inclined at an angle of 63.6 (1)°. The mol­ecular structure features short intra­molecular C—H⋯O contacts and the crystal packing exhibits weak inter­molecular C—H⋯S and π–π inter­actions [centroid-to-centroid distances 3.734 (2)–3.888 (2) Å].

## Related literature

For biological activities of carbazole derivatives, see: Diaz *et al.* (2002 ▶); Itoigawa *et al.* (2000 ▶); Ramsewak *et al.* (1999 ▶); Tachibana *et al.* (2001 ▶); Zhang *et al.* (2004 ▶). For the structures of closely related compounds, see: Chakkaravarthi *et al.* (2008*a* ▶,*b* ▶); Hökelek *et al.* (1998 ▶). For bond-length data, see: Allen *et al.* (1987 ▶). For graph-set notation, see: Etter *et al.* (1990 ▶). For general background, see: Govindasamy *et al.* (1998 ▶).
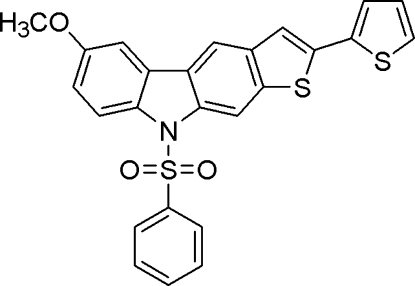


## Experimental

### 

#### Crystal data

C_25_H_17_NO_3_S_3_
*M* *_r_* = 475.58
Orthorhombic, 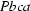

*a* = 15.3900 (12) Å
*b* = 10.1269 (7) Å
*c* = 28.233 (2) Å
*V* = 4400.2 (6) Å^3^
*Z* = 8
Mo *K*α radiation
μ = 0.37 mm^−1^
*T* = 295 (2) K
0.25 × 0.20 × 0.20 mm

#### Data collection

Bruker Kappa APEX2 diffractometer
Absorption correction: multi-scan (*SADABS*; Sheldrick, 1996 ▶) *T*_min_ = 0.914, *T*_max_ = 0.931
25628 measured reflections
5212 independent reflections
3570 reflections with *I* > 2σ(*I*)
*R*_int_ = 0.040

#### Refinement

*R*[*F* ^2^ > 2σ(*F* ^2^)] = 0.053
*wR*(*F* ^2^) = 0.175
*S* = 1.04
5212 reflections
290 parameters
2 restraints
H-atom parameters constrained
Δρ_max_ = 0.55 e Å^−3^
Δρ_min_ = −0.55 e Å^−3^

### 

Data collection: *APEX2* (Bruker, 2004 ▶); cell refinement: *SAINT* (Bruker, 2004 ▶); data reduction: *SAINT*; program(s) used to solve structure: *SHELXS97* (Sheldrick, 2008 ▶); program(s) used to refine structure: *SHELXL97* (Sheldrick, 2008 ▶); molecular graphics: *PLATON* (Spek, 2003 ▶); software used to prepare material for publication: *SHELXL97*.

## Supplementary Material

Crystal structure: contains datablocks I, global. DOI: 10.1107/S1600536809003493/bt2862sup1.cif

Structure factors: contains datablocks I. DOI: 10.1107/S1600536809003493/bt2862Isup2.hkl

Additional supplementary materials:  crystallographic information; 3D view; checkCIF report

## Figures and Tables

**Table 1:** Hydrogen-bond geometry (Å, °)

*D*—H⋯*A*	*D*—H	H⋯*A*	*D*⋯*A*	*D*—H⋯*A*
C6—H6⋯O1	0.93	2.57	2.930 (4)	103
C8—H8⋯O1	0.93	2.39	2.940 (3)	117
C19—H19⋯O2	0.93	2.39	2.943 (4)	118
C22—H22⋯S2^i^	0.93	2.80	3.684 (3)	158

Symmetry code: (i) 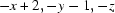.

**Fig. 1.**
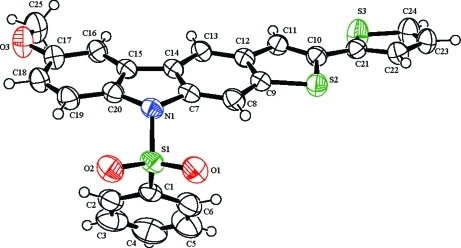
The molecular structure of the title compound, with atom labels and 50% probability displacement ellipsoids for non-H atoms.

**Fig. 2.**
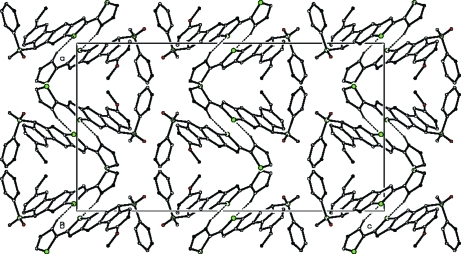
The packing of the title compound, viewed down the b axis. Intermolecular hydrogen bonds are shown as dashed lines. H atoms not involved in hydrogen bonding have been omitted.

## supplementary crystallographic information

### Comment

In continuation of our studies of carbazole derivatives, which are found to possess various biological activities, such as antitumor (Itoigawa *et al.*, 2000), antioxidative (Tachibana *et al.*, 2001), anti-inflammatory and antimutagenic (Ramsewak *et al.*, 1999), the crystal structure of the title compound has been determined. These compounds are thermally and photochemically stable, which makes them useful materials for technological applications. For instance, the carbazole ring is easily functionalized and covalently linked to other molecules (Diaz *et al.*, 2002). This enables its use as a convenient building block for the design and synthesis of molecular glasses, which are widely studied as components of electroactive and photoactive materials (Zhang *et al.*, 2004).

The geometric parameters of the title molecule (Fig. 1) agree well with reported similar structures (Chakkaravarthi *et al.*, 2008*a*,*b*; Hökelek *et al.*, 1998). The mean planes of thieno[2,3-*b*]carbazole and phenyl rings are inclined at an angle of 63.6 (1)°. Thiophene ring C21/C22/C23/C24/S3 forms a dihedral angle of 49.2 (1)° with phenyl ring. The methoxy group is slightly twisted [torsion angle C16—C17—O3—C25 is 13.9 (5)°] out of the plane of the benzene ring C15–C20.

The N—C bond lengths, namely N1—C7 and N1—C20 [1.434 (4) and 1.450 (4) Å] deviate slightly from the normal mean value reported in the literature (Allen *et al.*, 1987). This indicates that the substitution of the phenylsulfonyl group at atom N1 results the lengthening of C—N bond lengths. This may be due to the electron-withdrawing character of the phenylsulfonyl group (Govindasamy *et al.*, 1998).

A distorted tetrahedral geometry around S1 is observed. The deviations being seen for the O—S—O [O1—S1—O2 119.3 (1)°] and O—S—N [O1—S1—N1 106.8 (1)°] angles. The widening of the angles may be due to repulsive interactions between the two short S═O bonds, similar to what is observed in similar structures (Chakkaravarthi *et al.*, 2008*a*,*b*). The sum of the bond angles around N1 [343.3 (2)°] indicate the *sp*^2^ hybridized state of the atom N1 in the molecule.

The torsion angles O1—S1—N1—C7 and O1—S1—C1—C6 [50.2 (2)° and -22.3 (3)°, respectively] describe the *syn* conformation of the phenylsulfonyl group with respect to carbazole ring system. This conformation is influenced by the intramolecular C—H··· O hydrogen bonds (Table 1); C6—H6··· O1, C8—H8··· O1 and C19—H19···O2, involving sulfonyl atoms O1 and O2. In addition, intramolecular C8—H8···O1 and C19—H19···O2 hydrogen bonds form six-membered rings, both with a graph-set motif of *S*(6) and C19—H19···O2 forms five-membered ring, with graph-set motif of *S(5)*. The intermolecular C22—H22—S2 interaction generates a ten-membered ring, with graph-set motif of *R*_2_^2^(10) (Etter *et al.*, 1990).

The crystal structure of the title compound is stabilized by weak intermolecular C—H···S (Fig. 2 and Table 1) and π–π [*Cg*1···*Cg*3(2 - *x*, -*y*, -*z*) distance of 3.781 (2) Å; *Cg*1···*Cg*5(2 - *x*, -*y*, -*z*) distance of 3.734 (2) Å; *Cg*2···*Cg*6(2 - *x*, -*y*, -*z*) distance of 3.888 (2) Å, *Cg*3···*Cg*1(2 - *x*, -*y*, -*z*) distance of 3.781 (2) Å, *Cg*5···*Cg*5(2 - *x*, -*y*, -*z*) distance of 3.770 (2) Å and *Cg*6···*Cg*2(1 - *x*, 1 - *y*, -*z*) distance of 3.888 (2) Å (*Cg*1, *Cg*2, *Cg*3, *Cg*5 and *Cg*6 are the centroid of the rings defined by the atoms S2/C9/C12/C11/C10, S3/C21/C22/C23/C24, N1/C7/C14/C15/C20, C7/C8/C9/C12/C13/C14 and C15–C20, respectively)] interactions.

### Experimental

To a solution of diethyl-2-((2-bromomethyl)-5-methoxy-1- (phenylsulfonyl)-1*H*-indol-3-yl)methylene)malonate (0.2 g, 0.36 mmol) in dry 1,2-DCE (8 ml), ZnBr_2_ (0.16 g, 0.71 mmol) and bithiophene (0.07 g, 0.42 mmol) were added. The reaction mixture was then refluxed for 2 h under N_2_ atmosphere. It was then poured over ice–water (50 ml) containing 2 ml of conc. HCl, extracted with chloroform (3 × 10 ml) and dried (Na_2_SO_4_). The removal of solvent followed by flash column chromatographic purification (silica gel, 230–420 mesh, n-hexane/ethyl acetate 99:1) afforded the compound (I), suitable for X-ray analysis..

### Refinement

H atoms were positioned geometrically and refined using riding model with C—H = 0.93 Å and *U*_iso_(H) = 1.2Ueq(C) for aromatic H atoms and C—H = 0.96 Å and *U*_iso_(H) = 1.5Ueq(C) for methyl H atoms. The components of the anisotropic displacement parameters in direction of the bond of C21, C22, C23 and S3 were restrained to be equal within an effective standard deviation of 0.001 using the DELU command in SHELXL (Sheldrick, 2008).

### Crystal data

**Table:**

C_25_H_17_NO_3_S_3_	*F*(000) = 1968
*M**_r_* = 475.58	*D*_x_ = 1.436 Mg m^−^^3^
Orthorhombic, *P**b**c**a*	Mo *K*α radiation, λ = 0.71073 Å
Hall symbol: -P 2ac 2ab	Cell parameters from 6275 reflections
*a* = 15.3900 (12) Å	θ = 2.5–25.4°
*b* = 10.1269 (7) Å	µ = 0.37 mm^−^^1^
*c* = 28.233 (2) Å	*T* = 295 K
*V* = 4400.2 (6) Å^3^	Block, colourless
*Z* = 8	0.25 × 0.20 × 0.20 mm

### Data collection

**Table:**

Bruker Kappa APEX2 diffractometer	5212 independent reflections
Radiation source: fine-focus sealed tube	3570 reflections with *I* > 2σ(*I*)
graphite	*R*_int_ = 0.040
ω and φ scans	θ_max_ = 27.8°, θ_min_ = 1.4°
Absorption correction: multi-scan (*SADABS*; Sheldrick, 1996)	*h* = −17→20
*T*_min_ = 0.914, *T*_max_ = 0.931	*k* = −7→13
25628 measured reflections	*l* = −37→36

### Refinement

**Table:**

Refinement on *F*^2^	Primary atom site location: structure-invariant direct methods
Least-squares matrix: full	Secondary atom site location: difference Fourier map
*R*[*F*^2^ > 2σ(*F*^2^)] = 0.053	Hydrogen site location: inferred from neighbouring sites
*wR*(*F*^2^) = 0.175	H-atom parameters constrained
*S* = 1.04	*w* = 1/[σ^2^(*F*_o_^2^) + (0.0908*P*)^2^ + 2.7061*P*] where *P* = (*F*_o_^2^ + 2*F*_c_^2^)/3
5212 reflections	(Δ/σ)_max_ < 0.001
290 parameters	Δρ_max_ = 0.55 e Å^−^^3^
2 restraints	Δρ_min_ = −0.55 e Å^−^^3^

### Fractional atomic coordinates and isotropic or equivalent isotropic displacement parameters (Å^2^)

**Table:**

	*x*	*y*	*z*	*U*_iso_*/*U*_eq_	
C1	0.93528 (19)	−0.0624 (3)	0.20557 (10)	0.0443 (6)	
C2	0.8927 (2)	0.0321 (4)	0.23253 (11)	0.0603 (9)	
H2	0.9211	0.1084	0.2423	0.072*	
C3	0.8070 (3)	0.0097 (5)	0.24438 (14)	0.0821 (13)	
H3	0.7772	0.0714	0.2625	0.099*	
C4	0.7651 (3)	−0.1032 (6)	0.22961 (16)	0.0911 (14)	
H4	0.7071	−0.1165	0.2376	0.109*	
C5	0.8074 (3)	−0.1944 (5)	0.20365 (15)	0.0873 (14)	
H5	0.7784	−0.2704	0.1940	0.105*	
C6	0.8939 (2)	−0.1764 (4)	0.19114 (12)	0.0632 (9)	
H6	0.9232	−0.2397	0.1734	0.076*	
C7	0.98916 (17)	−0.0076 (3)	0.09633 (9)	0.0385 (6)	
C8	1.00292 (18)	−0.1328 (3)	0.07857 (10)	0.0447 (7)	
H8	1.0426	−0.1908	0.0921	0.054*	
C9	0.95349 (18)	−0.1667 (3)	0.03908 (9)	0.0404 (6)	
C10	0.87808 (19)	−0.2665 (3)	−0.03036 (10)	0.0432 (6)	
C11	0.85167 (18)	−0.1409 (3)	−0.02240 (10)	0.0434 (6)	
H11	0.8101	−0.0985	−0.0408	0.052*	
C12	0.89407 (17)	−0.0797 (3)	0.01716 (9)	0.0403 (6)	
C13	0.88459 (18)	0.0474 (3)	0.03532 (9)	0.0415 (6)	
H13	0.8474	0.1075	0.0208	0.050*	
C14	0.93126 (16)	0.0831 (3)	0.07524 (9)	0.0379 (6)	
C15	0.93339 (17)	0.2031 (3)	0.10301 (9)	0.0395 (6)	
C16	0.88881 (19)	0.3216 (3)	0.09834 (10)	0.0459 (7)	
H16	0.8497	0.3349	0.0737	0.055*	
C17	0.9044 (2)	0.4192 (3)	0.13154 (11)	0.0491 (7)	
C18	0.9644 (2)	0.4001 (3)	0.16782 (11)	0.0528 (8)	
H18	0.9740	0.4676	0.1896	0.063*	
C19	1.0099 (2)	0.2840 (3)	0.17224 (10)	0.0501 (7)	
H19	1.0509	0.2723	0.1961	0.060*	
C20	0.99236 (17)	0.1851 (3)	0.13975 (9)	0.0417 (6)	
C21	0.85065 (19)	−0.3600 (3)	−0.06662 (10)	0.0450 (6)	
C22	0.89216 (19)	−0.4794 (3)	−0.08041 (10)	0.0460 (6)	
H22	0.9418	−0.5142	−0.0664	0.055*	
C23	0.8466 (2)	−0.5371 (3)	−0.11860 (12)	0.0569 (8)	
H23	0.8657	−0.6131	−0.1339	0.068*	
C24	0.7753 (2)	−0.4750 (4)	−0.13083 (13)	0.0664 (9)	
H24	0.7385	−0.5036	−0.1548	0.080*	
C25	0.7922 (3)	0.5579 (4)	0.10138 (15)	0.0769 (11)	
H25A	0.8090	0.5441	0.0690	0.115*	
H25B	0.7699	0.6459	0.1050	0.115*	
H25C	0.7481	0.4953	0.1099	0.115*	
N1	1.02958 (15)	0.0539 (2)	0.13643 (8)	0.0421 (5)	
O1	1.08087 (14)	−0.1554 (2)	0.17329 (8)	0.0562 (6)	
O2	1.08494 (15)	0.0508 (2)	0.21908 (8)	0.0604 (6)	
O3	0.86577 (17)	0.5408 (2)	0.13127 (9)	0.0695 (7)	
S1	1.04166 (5)	−0.03348 (8)	0.18631 (2)	0.0445 (2)	
S2	0.95684 (5)	−0.31754 (8)	0.01006 (3)	0.0507 (2)	
S3	0.75741 (7)	−0.33734 (10)	−0.09832 (4)	0.0750 (3)	

### Atomic displacement parameters (Å^2^)

**Table:**

	*U*^11^	*U*^22^	*U*^33^	*U*^12^	*U*^13^	*U*^23^
C1	0.0485 (16)	0.0515 (16)	0.0329 (13)	0.0051 (13)	−0.0020 (11)	0.0074 (12)
C2	0.073 (2)	0.063 (2)	0.0449 (17)	0.0128 (17)	0.0092 (16)	0.0052 (15)
C3	0.085 (3)	0.096 (3)	0.065 (2)	0.036 (3)	0.031 (2)	0.021 (2)
C4	0.060 (2)	0.137 (4)	0.076 (3)	−0.010 (3)	0.019 (2)	0.016 (3)
C5	0.074 (3)	0.118 (4)	0.070 (3)	−0.037 (3)	0.020 (2)	−0.010 (2)
C6	0.067 (2)	0.073 (2)	0.0502 (18)	−0.0099 (18)	0.0100 (16)	−0.0031 (16)
C7	0.0345 (12)	0.0513 (16)	0.0297 (12)	−0.0015 (11)	0.0001 (10)	0.0009 (11)
C8	0.0442 (16)	0.0526 (17)	0.0372 (14)	0.0062 (13)	−0.0056 (12)	−0.0003 (12)
C9	0.0427 (14)	0.0433 (15)	0.0353 (13)	0.0000 (11)	0.0009 (11)	0.0000 (11)
C10	0.0455 (15)	0.0455 (15)	0.0386 (14)	−0.0005 (12)	−0.0039 (11)	0.0001 (12)
C11	0.0440 (15)	0.0464 (15)	0.0397 (14)	0.0008 (12)	−0.0085 (12)	0.0007 (12)
C12	0.0392 (14)	0.0467 (15)	0.0349 (13)	−0.0013 (11)	−0.0007 (11)	0.0003 (11)
C13	0.0398 (14)	0.0472 (15)	0.0376 (13)	0.0036 (12)	−0.0050 (11)	−0.0018 (12)
C14	0.0347 (13)	0.0459 (15)	0.0331 (13)	−0.0014 (11)	0.0012 (10)	−0.0010 (11)
C15	0.0373 (13)	0.0477 (15)	0.0335 (13)	−0.0042 (11)	0.0007 (10)	−0.0019 (11)
C16	0.0427 (15)	0.0526 (17)	0.0425 (15)	−0.0014 (13)	−0.0065 (12)	−0.0058 (13)
C17	0.0470 (15)	0.0495 (17)	0.0508 (17)	0.0020 (13)	−0.0015 (13)	−0.0084 (13)
C18	0.0599 (19)	0.0549 (18)	0.0437 (16)	−0.0056 (15)	−0.0068 (14)	−0.0108 (14)
C19	0.0535 (17)	0.0579 (18)	0.0388 (15)	−0.0058 (14)	−0.0109 (13)	−0.0043 (13)
C20	0.0381 (14)	0.0500 (16)	0.0370 (14)	−0.0040 (12)	−0.0011 (11)	−0.0007 (12)
C21	0.0501 (14)	0.0442 (15)	0.0407 (14)	−0.0049 (12)	−0.0022 (10)	0.0010 (12)
C22	0.0426 (15)	0.0519 (17)	0.0434 (15)	0.0052 (12)	−0.0069 (11)	−0.0041 (12)
C23	0.065 (2)	0.0474 (17)	0.0579 (18)	−0.0049 (15)	−0.0049 (14)	−0.0143 (14)
C24	0.067 (2)	0.065 (2)	0.067 (2)	−0.0017 (18)	−0.0189 (17)	−0.0188 (18)
C25	0.080 (3)	0.068 (2)	0.082 (3)	0.024 (2)	−0.022 (2)	−0.013 (2)
N1	0.0422 (12)	0.0502 (14)	0.0337 (11)	−0.0010 (10)	−0.0041 (9)	0.0006 (10)
O1	0.0533 (13)	0.0639 (13)	0.0514 (12)	0.0212 (11)	−0.0061 (10)	−0.0004 (10)
O2	0.0627 (14)	0.0726 (15)	0.0460 (12)	−0.0020 (11)	−0.0226 (10)	−0.0019 (11)
O3	0.0739 (16)	0.0571 (14)	0.0775 (17)	0.0132 (12)	−0.0235 (13)	−0.0201 (12)
S1	0.0414 (4)	0.0569 (5)	0.0353 (4)	0.0058 (3)	−0.0090 (3)	0.0010 (3)
S2	0.0622 (5)	0.0464 (4)	0.0435 (4)	0.0082 (3)	−0.0120 (3)	−0.0020 (3)
S3	0.0713 (6)	0.0670 (6)	0.0867 (7)	0.0164 (5)	−0.0298 (5)	−0.0227 (5)

### Geometric parameters (Å, °)

**Table:**

C1—C6	1.380 (5)	C14—C15	1.446 (4)
C1—C2	1.388 (4)	C15—C16	1.389 (4)
C1—S1	1.750 (3)	C15—C20	1.390 (4)
C2—C3	1.379 (6)	C16—C17	1.383 (4)
C2—H2	0.9300	C16—H16	0.9300
C3—C4	1.376 (7)	C17—O3	1.368 (4)
C3—H3	0.9300	C17—C18	1.393 (4)
C4—C5	1.347 (7)	C18—C19	1.374 (5)
C4—H4	0.9300	C18—H18	0.9300
C5—C6	1.388 (5)	C19—C20	1.385 (4)
C5—H5	0.9300	C19—H19	0.9300
C6—H6	0.9300	C20—N1	1.449 (4)
C7—C8	1.379 (4)	C21—C22	1.422 (4)
C7—C14	1.411 (4)	C21—S3	1.707 (3)
C7—N1	1.434 (3)	C22—C23	1.413 (4)
C8—C9	1.393 (4)	C22—H22	0.9300
C8—H8	0.9300	C23—C24	1.311 (5)
C9—C12	1.412 (4)	C23—H23	0.9300
C9—S2	1.734 (3)	C24—S3	1.692 (4)
C10—C11	1.354 (4)	C24—H24	0.9300
C10—C21	1.457 (4)	C25—O3	1.422 (4)
C10—S2	1.743 (3)	C25—H25A	0.9600
C11—C12	1.434 (4)	C25—H25B	0.9600
C11—H11	0.9300	C25—H25C	0.9600
C12—C13	1.394 (4)	N1—S1	1.674 (2)
C13—C14	1.384 (4)	O1—S1	1.422 (2)
C13—H13	0.9300	O2—S1	1.424 (2)
			
C6—C1—C2	121.4 (3)	C17—C16—C15	117.9 (3)
C6—C1—S1	118.7 (2)	C17—C16—H16	121.1
C2—C1—S1	119.8 (3)	C15—C16—H16	121.1
C3—C2—C1	118.1 (4)	O3—C17—C16	124.4 (3)
C3—C2—H2	121.0	O3—C17—C18	114.6 (3)
C1—C2—H2	121.0	C16—C17—C18	121.0 (3)
C4—C3—C2	120.8 (4)	C19—C18—C17	121.5 (3)
C4—C3—H3	119.6	C19—C18—H18	119.2
C2—C3—H3	119.6	C17—C18—H18	119.2
C5—C4—C3	120.5 (4)	C18—C19—C20	117.4 (3)
C5—C4—H4	119.7	C18—C19—H19	121.3
C3—C4—H4	119.7	C20—C19—H19	121.3
C4—C5—C6	120.8 (4)	C19—C20—C15	121.8 (3)
C4—C5—H5	119.6	C19—C20—N1	128.9 (3)
C6—C5—H5	119.6	C15—C20—N1	109.3 (2)
C1—C6—C5	118.5 (4)	C22—C21—C10	127.9 (3)
C1—C6—H6	120.7	C22—C21—S3	110.4 (2)
C5—C6—H6	120.7	C10—C21—S3	121.7 (2)
C8—C7—C14	122.8 (2)	C23—C22—C21	109.7 (3)
C8—C7—N1	128.3 (2)	C23—C22—H22	125.1
C14—C7—N1	108.9 (2)	C21—C22—H22	125.1
C7—C8—C9	115.7 (3)	C24—C23—C22	114.7 (3)
C7—C8—H8	122.2	C24—C23—H23	122.7
C9—C8—H8	122.2	C22—C23—H23	122.7
C8—C9—C12	123.4 (3)	C23—C24—S3	112.9 (3)
C8—C9—S2	125.4 (2)	C23—C24—H24	123.6
C12—C9—S2	111.2 (2)	S3—C24—H24	123.6
C11—C10—C21	129.8 (3)	O3—C25—H25A	109.5
C11—C10—S2	112.2 (2)	O3—C25—H25B	109.5
C21—C10—S2	118.0 (2)	H25A—C25—H25B	109.5
C10—C11—C12	113.5 (3)	O3—C25—H25C	109.5
C10—C11—H11	123.3	H25A—C25—H25C	109.5
C12—C11—H11	123.3	H25B—C25—H25C	109.5
C13—C12—C9	118.9 (2)	C7—N1—C20	106.1 (2)
C13—C12—C11	129.6 (3)	C7—N1—S1	118.85 (19)
C9—C12—C11	111.5 (2)	C20—N1—S1	118.30 (18)
C14—C13—C12	119.1 (3)	C17—O3—C25	117.3 (3)
C14—C13—H13	120.5	O1—S1—O2	119.31 (14)
C12—C13—H13	120.5	O1—S1—N1	106.77 (13)
C13—C14—C7	120.1 (3)	O2—S1—N1	106.37 (13)
C13—C14—C15	132.2 (3)	O1—S1—C1	109.39 (15)
C7—C14—C15	107.7 (2)	O2—S1—C1	109.64 (15)
C16—C15—C20	120.4 (3)	N1—S1—C1	104.24 (12)
C16—C15—C14	131.6 (2)	C9—S2—C10	91.58 (13)
C20—C15—C14	108.0 (2)	C24—S3—C21	92.11 (16)
			
C6—C1—C2—C3	−0.5 (5)	C16—C15—C20—C19	−1.1 (4)
S1—C1—C2—C3	175.8 (3)	C14—C15—C20—C19	179.0 (3)
C1—C2—C3—C4	−0.3 (6)	C16—C15—C20—N1	−179.9 (2)
C2—C3—C4—C5	0.7 (7)	C14—C15—C20—N1	0.2 (3)
C3—C4—C5—C6	−0.2 (7)	C11—C10—C21—C22	165.1 (3)
C2—C1—C6—C5	0.8 (5)	S2—C10—C21—C22	−14.8 (4)
S1—C1—C6—C5	−175.4 (3)	C11—C10—C21—S3	−16.4 (5)
C4—C5—C6—C1	−0.5 (6)	S2—C10—C21—S3	163.72 (17)
C14—C7—C8—C9	−2.2 (4)	C10—C21—C22—C23	−177.0 (3)
N1—C7—C8—C9	179.5 (3)	S3—C21—C22—C23	4.4 (3)
C7—C8—C9—C12	1.4 (4)	C21—C22—C23—C24	−3.9 (4)
C7—C8—C9—S2	−179.3 (2)	C22—C23—C24—S3	1.7 (4)
C21—C10—C11—C12	179.9 (3)	C8—C7—N1—C20	179.9 (3)
S2—C10—C11—C12	−0.2 (3)	C14—C7—N1—C20	1.4 (3)
C8—C9—C12—C13	0.8 (4)	C8—C7—N1—S1	−43.8 (4)
S2—C9—C12—C13	−178.6 (2)	C14—C7—N1—S1	137.7 (2)
C8—C9—C12—C11	−179.5 (3)	C19—C20—N1—C7	−179.6 (3)
S2—C9—C12—C11	1.2 (3)	C15—C20—N1—C7	−1.0 (3)
C10—C11—C12—C13	179.0 (3)	C19—C20—N1—S1	43.8 (4)
C10—C11—C12—C9	−0.7 (4)	C15—C20—N1—S1	−137.5 (2)
C9—C12—C13—C14	−2.3 (4)	C16—C17—O3—C25	13.9 (5)
C11—C12—C13—C14	178.0 (3)	C18—C17—O3—C25	−167.9 (3)
C12—C13—C14—C7	1.6 (4)	C7—N1—S1—O1	50.2 (2)
C12—C13—C14—C15	−177.5 (3)	C20—N1—S1—O1	−178.7 (2)
C8—C7—C14—C13	0.7 (4)	C7—N1—S1—O2	178.7 (2)
N1—C7—C14—C13	179.3 (2)	C20—N1—S1—O2	−50.3 (2)
C8—C7—C14—C15	−179.9 (2)	C7—N1—S1—C1	−65.5 (2)
N1—C7—C14—C15	−1.3 (3)	C20—N1—S1—C1	65.6 (2)
C13—C14—C15—C16	0.0 (5)	C6—C1—S1—O1	−22.2 (3)
C7—C14—C15—C16	−179.2 (3)	C2—C1—S1—O1	161.4 (2)
C13—C14—C15—C20	179.9 (3)	C6—C1—S1—O2	−154.8 (3)
C7—C14—C15—C20	0.7 (3)	C2—C1—S1—O2	28.9 (3)
C20—C15—C16—C17	−0.7 (4)	C6—C1—S1—N1	91.6 (3)
C14—C15—C16—C17	179.1 (3)	C2—C1—S1—N1	−84.7 (3)
C15—C16—C17—O3	179.6 (3)	C8—C9—S2—C10	179.6 (3)
C15—C16—C17—C18	1.5 (5)	C12—C9—S2—C10	−1.1 (2)
O3—C17—C18—C19	−178.7 (3)	C11—C10—S2—C9	0.7 (2)
C16—C17—C18—C19	−0.4 (5)	C21—C10—S2—C9	−179.4 (2)
C17—C18—C19—C20	−1.4 (5)	C23—C24—S3—C21	0.9 (3)
C18—C19—C20—C15	2.2 (4)	C22—C21—S3—C24	−3.1 (3)
C18—C19—C20—N1	−179.3 (3)	C10—C21—S3—C24	178.2 (3)

### Hydrogen-bond geometry (Å, °)

**Table:**

*D*—H···*A*	*D*—H	H···*A*	*D*···*A*	*D*—H···*A*
C6—H6···O1	0.93	2.57	2.930 (4)	103
C8—H8···O1	0.93	2.39	2.940 (3)	117
C19—H19···O2	0.93	2.39	2.943 (4)	118
C22—H22···S2^i^	0.93	2.80	3.684 (3)	158

Symmetry codes: (i) −*x*+2, −*y*−1, −*z*.
